# Effect of a Traction Exercise Neck Brace on Cervical Spondylopathy Radiculopathy: A Clinical Study and Finite Element Analysis

**DOI:** 10.1155/2021/8825150

**Published:** 2021-04-13

**Authors:** Liang-Xing Xiao, Chang-Shun Liu, Shi-Zhen Zhong, Wen-Hua Huang

**Affiliations:** ^1^School of Basic Medical Sciences, Southern Medical University, Guangzhou 510515, China; ^2^Foshan Yitai Medical Supplies Co., Ltd, Foshan 528200, China; ^3^School of Traditional Chinese Medicine, Southern Medical University, Guangzhou 510515, China

## Abstract

Traction of cervical spine is an effective method for the treatment of cervical spondylotic radiculopathy (CSR). In this study, a cervical tractor named traction exercise neck brace (TENB) was used to evaluate its effect on the patients with CSR. Forty CSR volunteers were recruited and randomly divided into two groups. One group was subjected to cervical muscle exercise with TENB under static traction condition. Another group was subjected to (JOBT) as controls. Symptoms of CSR were evaluated by the visual analogue scale (VAS) and neck disability index (NDI). Imaging characteristics were assessed by curvature of the cervical spine and size of the intervertebral foramen. A finite element (FE) analysis model of cervical spine was established by 3D reconstruction to simulate the TENB traction, which evaluates the biomechanical performance. Results showed that TENB significantly reduced scores of VAS and NDI in subjects, and this improved effect on symptoms of pain and radiculopathy is better than that of JOBT. TENB also improved the cervical curvature and enlarged intervertebral foramen at the C4–C6 level. Moreover, FE analysis found that simulated TENB traction increased the spacing of intervertebral foramen, intervertebral disc, and zygapophyseal and uncovertebral joints and changed the stress distribution on the facet joints and nucleus pulposus. This study demonstrates that TENB relieves the symptoms of CSR by adjusting structure of cervical vertebra and restoring its biomechanical performance, which may be a promising instrument in the treatment of CSR.

## 1. Introduction

Cervical spondylotic radiculopathy (CSR), accounting for approximately 60% of all the cervical spondylosis (CS), is caused by compression or irritation of cervical nerve root [1, 2]. Clinical features of CSR include neck and scapular pain radiating from the neck into the distribution fractions of the affected root involving arms and fingers [1, 3]. In recent years, the incidence of CSR increases gradually and presents a younger trend; its disorder seriously impacts the life quality of patients [2]. Two common causes for CSR are pathological degenerative changes in the uncovertebral and zygapophyseal joints and herniation of intervertebral discs, which results in the foraminal encroachment of spinal nerve [3–5].

Conservative management is recommended to be the initial therapy choice for most patients with CSR, such as medication, traditional Chinese medicine, and traction of the cervical spine. These treatments attenuate symptoms of CSR mainly by relieving neck pain and improving cervical function, but they have their disadvantages. For example, medication using anti-inflammatory drugs [6] or epidural steroid injection [7] cannot alleviate the mechanical compression of nerve root. Manipulation and acupuncture cannot consolidate the curative effect, only relieving the pain in a short term [8, 9]. Conventional cervical traction like jaw-occipital belt traction (JOBT) reduces the compliance of patients due to its long course of treatment and being performed in the hospital [10]. Currently, in order to improve the compliance of therapy so that the patient could be willing to receive treatment for a long time, portable home spinal traction devices for CSR were developed, while the insufficient clinical efficacy of these devices limits their application [11, 12]. Therefore, it is necessary to develop an apparatus for cervical traction that features satisfactory efficacy and safety and better compliance, which may be helpful for the conservative treatment of CSR.

**Figure 1:**
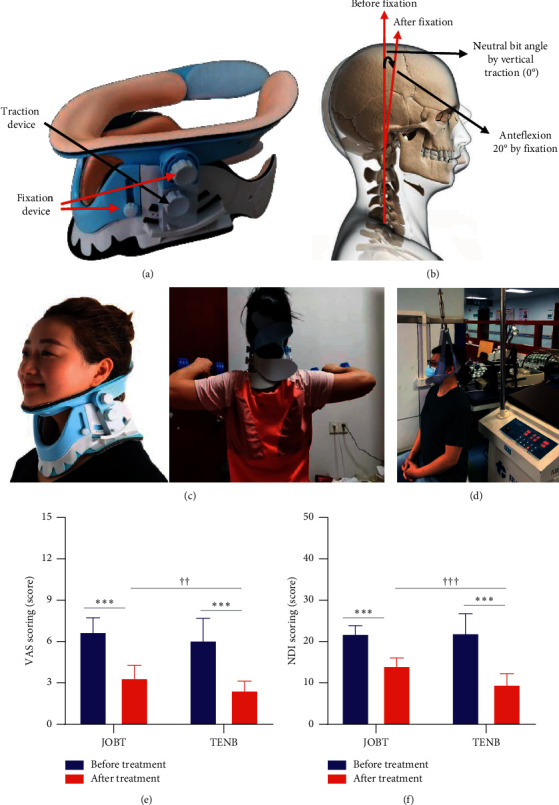
Effect of TENB on the symptoms of patients with CSR. (a) TENB. (b) Fixation of head and neck posture for vertical traction. (c) Experiment traction by TENB. (d) Conventional traction by JOBT. (e) VAS scores. (f) NDI scores. ^*∗∗∗*^*P* < 0.001, vs. before treatment; ^††^*P* < 0.01; ^†††^*P* < 0.001, TENB vs. JOBT.

In our early works, a cervical tractor named traction exercise neck brace (TENB; Figure 1(a)) was developed for the treatment of CSR. According to our design, TENB is composed of fixation and traction structures. It is capable of traction of cervical spine after fixing the posture of head and neck; meanwhile, subjects can also perform muscle resistance exercise under static traction, which relives the symptoms of CSR. In fact, cervical traction relates to the anteflexed posture of head and neck. With the anteflexed angles adjusted from 5° to 20°, the maximum traction tress concentrates from upper cervical vertebrae to the lower [13, 14]. In the CSR, the frequent lesion site is C5-C6 [15]; due to the structural features of cervical spine, when vertical traction is carried out with JOBT that lacks head and neck fixtures, the direction of traction force does not coincide with the normal line of cervical cross section. As a result, the maximum traction stress is not concentrated at the C5-C6. However, TENB can keep the traction angle in line with the direction of traction force by fixing the posture of head and neck to 20° of anteflexion, which improves the accuracy of traction (Figure 1(b)). In this regard, TENB may have more advantage in the treatment of CSR, in comparison with JOBT. However, the effect of TENB on CSR remains undetected.

In general, development of CSR changes biomechanical performance of the cervical spine due to its degenerative condition involving vertebral discs and adjacent structures. Biomechanical tests can evaluate severity of cervical lesion and efficacy and safety of traction intervention on the spine. However, it is very difficult to obtain the biomechanical data in an actual clinical setting. To study the impact of traction on the cervical vertebra, therefore, a finite element (FE) analysis model of spinal segments is appropriate in which the cervical dysfunctions in the pathologic degenerative condition can be simulated. This computational method provides insight into the detailed biomechanics of the human neck both with and without traction, revealing the impact of tractor on the inner workings of cervical spine [16, 17]. Therefore, in this study, the present work investigated the effects of TENB on CSR in the volunteers enrolled as compared with conventional therapy of JOBT. Moreover, biomechanical performance of TENB on cervical vertebra was explored in a FE model of cervical spine.

## 2. Data and Methods

This study was performed and approved by the Ethics Committee of Wuyi Hospital of Traditional Chinese Medicine (Jiangmen, China). All volunteers provided a written informed consent before initiation of the study.

### 2.1. Clinical Study

#### 2.1.1. Clinical Management

Forty patients with CSR (aged 21–51 years; median age 34.5 years) were recruited in the hospital between January 2019 and June 2020. Of them, 18 were male and 22 were female. They all presented with symptoms (numbness and pain) and signs of root distribution which conform to the diagnostic criteria of CSR. Computed tomography (CT) and magnetic resonance imaging examination showed C5/6 lateral protrusion, consistent with the clinical manifestations. However, cervical instability and disc calcification were not observed. Patients were randomly divided into two groups (*n* = 20). One group was subjected to cervical traction with TENB for 30 minutes at home. After fixing the posture of head and neck to 20° of anteflexion, for the first 10 minutes under vertical static traction, subjects stood and held 1.5 kg dumbbells and lifted them flat for chest expansion exercise for 1 minute and then put the arms down and placed over the thighs and rested for a minute, which is a group of muscle resistance exercises. A total of five groups of exercise were conducted. For the next 20 minutes, subjects sat down in the chair and were only subjected to vertical traction. This treatment was performed twice a day for 4 weeks (Figure 1(c)). Another group was subjected to jaw-occipital belt traction (JOBT) for vertical traction while sitting in the chair in hospital as controls (Figure 1(d)). Moreover, the height of chair used in this study is 45 cm, and the traction force is set according to the subject's endurance. Subjects remained relaxed while sitting down and were not allowed to support their torso using their hands. During the treatment, recruited patients did not receive other treatments.

### 2.2. Clinical Evaluation

**Figure 2:**
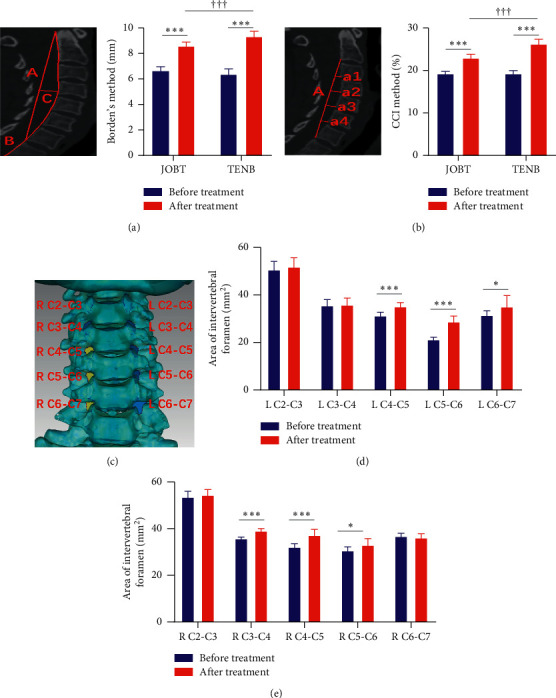
Effect of TENB on the curvature and intervertebral foramen of cervical vertebra. Measurements of cervical curvature by Borden (a) and CCI (b) methods. (c) A 3D model of cervical spine. Size of intervertebral foramen on the left (d) and right (e) sides in the TENB group. ^*∗*^*P* < 0.05; ^*∗∗∗*^*P* < 0.001, vs. before treatment; ^†††^*P* < 0.001, TENB vs. JOBT.

Clinical symptoms were evaluated by visual analogue scale (VAS) and neck disability index (NDI) as in the previous study [18]. Physiological curvature of cervical spine was evaluated using the method of Borden and cervical curvature index (CCI). By Borden's method, at the widest point between lines A and B, the length of vertical cross line (line C) is used to assess the curvature depth of cervical vertebra (Figure 2(a)). The calculation formula of CCI value is CCI = (*a*1 + *a*2 + *a*3 + *a*4)/*A* × 100%, reflecting the cervical curvature (Figure 2(b)). Moreover, the area of intervertebral foramen was measured by three-dimensional (3D) reconstruction of CT images of the cervical spine in Mimics Research 19.0 software. Then, the data were corrected to superpose the two adjacent pedicles in Geomagic Studio 2012 software. A plane was obtained by cutting the superposed pedicle center of the spinal canal, which defines the contour line of internal intervertebral foramen. The contour line data were imported into the Siemens NX 10.0 software to construct a bounded plane, which obtains the internal foraminal plane. Finally, the foraminal area between each vertebral body was measured by software analysis (Figure 2(c)).

### 2.3. Finite Element Modeling and Analysis

#### 2.3.1. Finite Element Modeling

Before treatment, thin-layer spiral CT scanning was carried out on the cervical vertebra of an enrolled female subject when she wore TENB. The posture of head and neck was fixed to keep the 20° of anteflexion when the patient wore TENB. This angle was taken as the neutral bit angle of traction force (0°) in the simulated traction. CT instrument scanned from the base of the occipital bone to the seventh cervical vertebra (C0–C7) with a layer thickness of 0.625 mm. All the original 2D CT scanning images were obtained and stored in the DICOM format for 3D reconstruction models. The original CT data in the DICOM format were input into the software (Mimics 21.0, Materialise, Leuven, Belgium) for 3D image reconstruction and output for mesh generation by HyperMesh 12.0 (Altair Company, USA). The material properties of various tissues were made homogeneous and isotropic, and ligaments and muscles were simulated with linear spring. Specific assignment parameters of the model are presented in Table 1, and the numbers of units and nodes of FE model are in Table 2.

#### 2.3.2. Model Validation

FE model was validated in different static loading conditions, and the predicted range of motion of each adjacent vertebra was compared against previous studies [19, 20]. In the FE model, all degrees of freedom were constrained on the lower surface of C7. Pure moments load of 1 Nm was applied in the three anatomical planes of model to simulate the flexion, extension, lateral bending, and axial rotation, respectively. Moreover, to simulate physical loading of head weight, a compressive follower load of 50 N was used to the upper surface of C0 [21]. The validation and FE analysis experiments were implemented in ABAQUS v6.9.1 (3DS, Waltham, MA).

#### 2.3.3. FE Analysis

**Figure 3:**
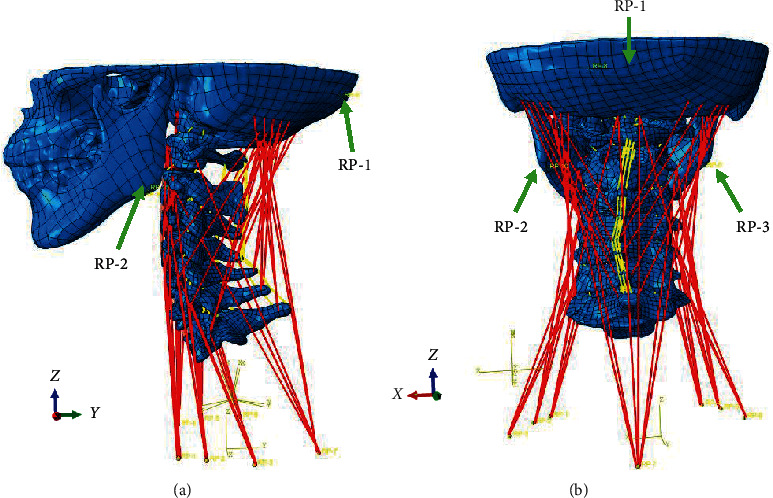
Three-dimensional FE model of intact cervical vertebra (C1–C7). (a) Lateral and (b) posterior views of the spine. RP-1, RP-2, and RP-3 indicate the force loading points that simulated the TENB traction.

An analysis of simulated TENB traction was performed in ABAQUS v6.9.1. All the nodes of C7 end plate in the FE model were restrained and its freedom degree of at the directions of *X*, *Y*, and *Z* was zero. The 3D model reconstructed from therapeutic position fixed with TENB was taken as the neutral bit angle of traction force (0°), and a 50 N of traction force was applied in the reference points corresponding to the occipital bone (RP-1) and two sides of the mandible (RP-2 and RP-3) (Figure 3). Similarly, other angles of traction force were determined, including anteflexion 20° (+20°) and 10° (+10°) and rear protraction 10° (−10°) and 15° (−15°). Changes in the biomechanics of cervical vertebra were measured and analyzed by the following indexes: (1) spacing of intervertebral foramen, (2) spacing and stress of zygapophyseal joint and uncovertebral joints, and (3) spacing and stress of intervertebral discs. Biomechanical changes of cervical vertebra before and after the simulative traction were measured through displacement difference by referencing the similar study [17]. For example, in the measurement of intervertebral foraminal spacing, one point was selected in the position of intervertebral foramen, and the coordinate values before and after the traction in the direction of *Z*-axis were recorded. After subtracting the coordinate value before the traction, displacement difference is obtained, which is the spacing of intervertebral foramen.

### 2.4. Statistical Analysis

Statistical analyses were conducted using SPSS 25.0 software. Normal distribution of the data was assessed by the Kolmogorov–Smirnov test. Comparisons of clinical symptoms evaluation, curvature of cervical vertebrae, and size of intervertebral foramen between groups were performed using a paired-samples *T* test or one-way analysis of variance. Differences were defined statistically significant at *P* < 0.05.

## 3. Results

### 3.1. Clinical Evaluation

#### 3.1.1. TENB Ameliorated the Symptoms of Subjects with CSR

After treatment, VAS scores in the TENB group decreased from 6.10 to 2.45 and NDI scores from 22.05 to 9.60 (Figures 1(e) and 1(f)), both showing a decrease by more than 50% (*P* < 0.001). Moreover, the values of VAS and NDI in the TENB group were lower than those in the JOBT group. These indicate that TENB significantly ameliorated the clinical symptoms of patients with CSR, and its efficacy was better than that of conventional JOBT.

#### 3.1.2. TENB Improved the Imaging Characteristics of Cervical Vertebra

The physiological curvature of cervical vertebra was measured using Borden and CCI methods. In the TENB group, the value measured by Borden's method increased from 6.36 to 9.32 after treatment (Figure 2(a)) and the value measured by CCI's method from 19.25 to 26.22 (Figure 2(b)). The same trends were also observed in the JOBT group after treatment, but the values in the former were significantly higher than in the latter (*P* < 0.001). These data imply that TENB may remarkably improve the curvature of cervical vertebra in patients with CSR and its efficacy may be better than that of JOBT.

The size of intervertebral foramen in the TENB group was detected through a 3D model of the cervical spine (Figure 2(c)). On the left side, the size of intervertebral foramen of C4–C7 was significantly enhanced after treatment (Figure 2(d)). On the right side, TENB treatment remarkably increased the size of intervertebral foramen of C3-6 (Figure 2(e)). These findings indicate that TENB treatment may increase the size of intervertebral foramen of C4-C5 and of C5-C6.

### 3.2. Biomechanics Analysis

Model validation assessments indicated that each index verified that the FE model established in this study was successful (Figure 3) so that it would be effective to analyze the biomechanical performance of cervical vertebra at different angles from the simulated traction force.

#### 3.2.1. Simulated Traction Adjusted the Structure of Cervical Vertebrae

**Figure 4:**
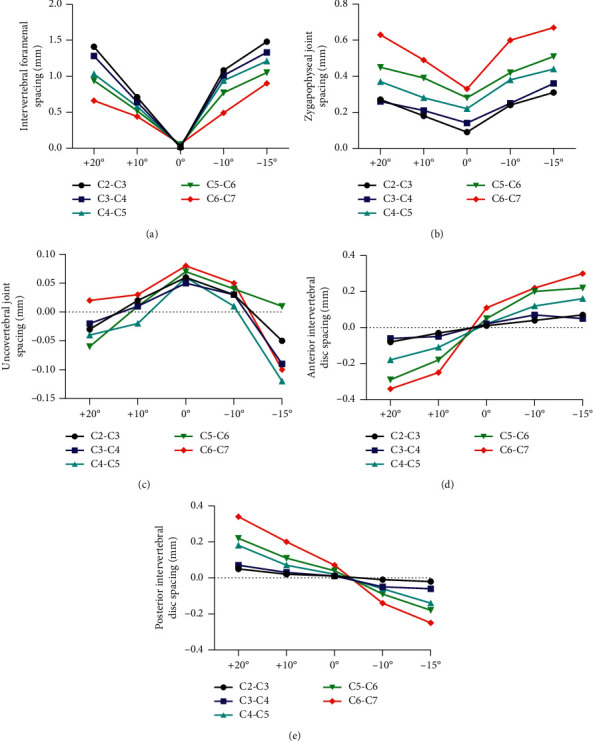
Effect of TENB on the structure of cervical vertebra. (a) Intervertebral foramen spacing. (b) Zygapophyseal joint spacing. (c) Uncovertebral joint spacing. (d) Anterior and (e) posterior intervertebral disc spacing. Angle values in the abscissa stand for the different angles of traction force.

The impact of TENB on the cervical vertebral structure was studied on the FE model. In both the anteflexion and rear protraction directions, FE traction increased the spacing of intervertebral foramen and zygapophyseal joint in an angle-dependent manner (Figures 4(a) and 4(b)). FE traction increased the uncovertebral joint space within a range from +10° to −10° (Figure 4(c)).

Simulated FE traction also changed the spacing of intervertebral disc. The spacing of anterior intervertebral disc was decreased in the anteflexion direction of traction force and increased in the rear protraction directions (Figure 4(d)). However, opposite tendency was found in the posterior intervertebral disc. FE traction enhanced the spacing of posterior intervertebral disc in the anteflexion directions but reduced the spacing in the rear protraction directions (Figure 4(e)).

#### 3.2.2. Simulated FE Traction Changed the Stress Distribution on Cervical Vertebra

**Figure 5:**
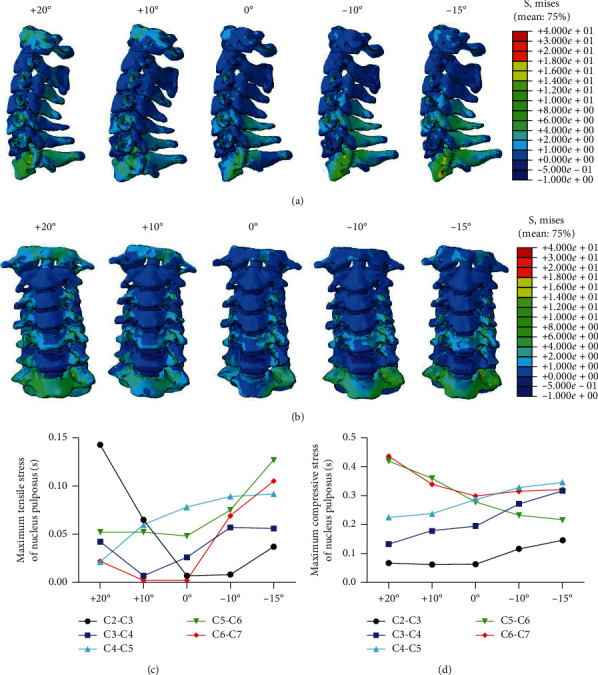
Internal von Mises stress on cervical vertebra at different simulated angles of traction force. (a) Lateral and (b) anterior views of cervical spine. (c) Maximum tensile stress and (d) maximum compressive stress of nucleus pulposus.

Simulated FE traction regulated the stress distribution on cervical vertebra, mainly in the lower cervical spine. The zygapophyseal joint and spinous process of C4–C7 showed a high-stress region in the rear protraction directions while no significant stress was found in the anteflexion direction (Figure 5(a)). Moreover, FE traction enhanced the stress on the uncovertebral joint of C5–C7 at the tested angles of traction force (Figure 5(b)).

In addition, simulated FE traction increased the stress of nucleus pulposus at the tested angles of traction force. FE traction enhanced the maximum tensile stress of nucleus pulposus of intervertebral disc at C4–C6 under the tested conditions (Figure 5(c)). Moreover, FE traction increased the maximum compressive stress of nucleus pulposus at all traction angles, and the stress values were increased gradually from C2-C3 to C6-C7 along the spine (Figure 5(c)).

## 4. Discussion

The present study carried out a clinical assessment of TENB in the subjects with CSR. The patients had varying degrees of relief from symptoms and a decrease in VAS and NDI scores, demonstrating a remarkable effect of TENB on CSR. This agrees with the previous report that traction ameliorated the symptoms of CSR [22]. In our study, traction with TENB was carried out after adjusting the head and neck posture to 20° of anteflexion. Results found that this manipulation concentrates the maximum stress of traction on the lower cervical spine, which may alleviate the compression of nerve root in subjects with CSR.

VAS and NDI scores are commonly used for pain assessment. Neck pain due to compression of nerve root is a major symptom of CSR which involves changes in the cervical vertebra and neck muscles. Our clinical study found that TENB decreased the VAS and NDI scores, indicating a relief of neck pain. TENB was designed mainly to resist head weight and separate cervical vertebral bodies and facet joints so as to decrease pressure on the discs or nerves [23] and improve the spinal curvature. Additionally, besides the traction function like JOBT, for the first 10 minutes, TENB treatment involved five groups of muscle resistance exercises under static traction. This may explain the result of our study that TENB had a better effect on relief of neck pain and regulation of cervical curvature than conventional JOBT did. Degenerative changes in the muscles attached to the cervical vertebra are also related with the loss of cervical curvature, resulting in weakening sternocleidomastoid, levator scapulae muscle, posterior cervical extension muscles, and upper trapezius, which aggravates the symptoms of CSR [18, 24]. In addition to adjusting spinal vertebra, TENB can force its wearers to exercise the neck and shoulder muscles by holding a weight in both hands, which significantly improves the cervical curvature and relieves neck pain.

Foraminal encroachment of spinal nerve is a major cause of CSR. Thus, the size of intervertebral foramen was measured to evaluate the effect of TENB on CSR. Results showed that TENB enlarged the intervertebral foraminal size mainly at the C4-C5 and C5-C6 levels. Our finding agrees with previous ones that traction increased the size and height of cervical neural foramen to relieve the nerve compression [25, 26]. As narrowing of nerve root canal is correlated with changes in the cervical biomechanics, we further investigated the biomechanical performance of TENB in a FE model. Results revealed that simulated traction increased the spacing of intervertebral foramen, consistent with our clinical data. Both our clinical and FE analysis experiments thus demonstrated that TENB was capable of increasing the neural foraminal size. Moreover, TENB traction increased the spacing of intervertebral discs and zygapophyseal and uncovertebral joints. In fact, degenerative changes in the nucleus pulposus not only induce formation of osteophyte, but also increase bearing stress around accessory facet joints which leads to development of osteophyte in the facet joints. Thus, degenerative conditions occur in the tissues surrounding the intervertebral foramen, including herniated nucleus pulposus, degenerative uncovertebral joints anteriorly, and hypertrophic facet joints posteriorly. These nodal points of pathogenesis further aggravate instability of the spinal segment, causing primary symptoms related to CSR [3–5]. Our study demonstrates that TENB increases the space of facet joints and the height of intervertebral discs, relieves stress on these tissues, and enhances the stability of cervical spine, thus alleviating compression of cervical nerve and symptoms of CSR.

Our FE analysis reveals the biomechanical mechanism of how TENB treats CSR. The following aspects should be taken into consideration in clinical application of TENB for CSR. (1) Indications. The most common segment affected by CSR is C5-6 (C6 nerve root), followed by C4-5 and C6-7 [15, 27]. Simulated traction improved the stress distribution in the zygapophyseal and uncovertebral joints and nucleus pulposus mainly at the C4–C7 and at the C5-C6 as well. These findings suggest that TENB may modulate the stress distribution on the cervical spine structures and indicate that TENB is effective for common CSR patients. (2) Safety. Traction increased the space of uncovertebral joints at 0° and decreased the apace at +20° and −15°. These findings imply that an excessive traction angle may induce instability of cervical spine. Thus, an appropriate angle of traction force should be within a range from +10 to −10° during the treatment. (3) Traction guidance for CSR. First, the maximum space of uncovertebral joint was shown at the traction force of 0°. This angle can be used for the patients with obvious hyperplasia of uncovertebral joints. Secondly, since distraction of the facets results in stabilization of spinal segments and enhances the space of spinal cord and root [28], it will be better to tract anteriorly within a range from 0° to 10° for the hypertrophic zygapophyseal joints. Thirdly, patients with an early stage of simple disc bulging may be subjected to posterior extensor traction of less than 10°, which helps return intervertebral disc, relieve muscle tension, and restore abnormal curvature. In the present study, TENB improved the symptoms of subjects with CSR by increasing the space of intervertebral foramen. Compared with common JOBT therapy, TENB treatment of CSR can not only regulate the cervical vertebra but also relax the muscles in the neck and shoulder. However, as the present study did not clarify how the TENB affects the condition of muscles and then improves the compression of spinal nerve, further study is warranted.

## 5. Conclusions

This study demonstrates that TENB treatment significantly improves the curvature of cervical spine and increases the size of intervertebral foramen, thus effectively ameliorating the symptoms of CSR. Moreover, FE analysis study shows that simulated traction increases the spacing of intervertebral foramen, intervertebral disc, and zygapophyseal and uncovertebral joints and changes the stress distribution on the facet joints and nucleus pulposus of cervical spine, indicating that TENB treatment of CSR may adjust the anatomical structure of cervical vertebra and improve the biomechanical performance of cervical vertebra. In consideration of both efficacy and safety of TENB for cervical vertebra, angles of traction force in the anteflexion and rear protraction directions should not exceed 10°. Taken together, TENB is helpful in treatment of CSR by regulating the structure of cervical vertebra and restoring its biomechanics.

## Figures and Tables

**Table 1:** Material properties of the FE model of cervical spine.

	Material	Elasticity modulus (MPa)	Poisson's ratio
Bone tissue	Cortical bone	11000	0.29
	Cancellous bone	500	0.29
	Articular cartilage	10	0.3
	Nucleus pulposus	1	0.49
	Fibrous ring	3.4	0.4
	End plate	600	0.4

Ligaments	Transverse ligament	20	0.25
	Other ligaments	10	0.3

Muscles	Various muscles	65	0.39

**Table 2:** Unit and node numbers in FE model.

	Material	Unit number	Node number
Cervical	C1	18896	66884
	C2	11215	37890
	C3	8580	28902
	C4	10887	37725
	C5	9049	27919
	C6	7487	33968
	C7	10078	31033

Disc	C2-C3	1702	4770
	C3-C4	1827	5197
	C4-C5	2687	8921
	C5-C6	1942	5887
	C6-C7	2360	7548

## Data Availability

The data used to support the findings of this study are available from the corresponding author upon request.
